# Near Infrared Photoimmunotherapy: A Review of Recent Progress and Their Target Molecules for Cancer Therapy

**DOI:** 10.3390/ijms24032655

**Published:** 2023-01-31

**Authors:** T. M. Mohiuddin, Chaoyu Zhang, Wenjie Sheng, Marwah Al-Rawe, Felix Zeppernick, Ivo Meinhold-Heerlein, Ahmad Fawzi Hussain

**Affiliations:** Department of Gynecology and Obstetrics, Medical Faculty, Justus-Liebig-University Giessen, Klinikstr. 33, 35392 Giessen, Germany

**Keywords:** cancer therapy, NIR-PIT, ICD, antitumor host immunity, target molecule

## Abstract

Near infrared photoimmunotherapy (NIR-PIT) is a newly developed molecular targeted cancer treatment, which selectively kills cancer cells or immune-regulatory cells and induces therapeutic host immune responses by administrating a cancer targeting moiety conjugated with IRdye700. The local exposure to near-infrared (NIR) light causes a photo-induced ligand release reaction, which causes damage to the target cell, resulting in immunogenic cell death (ICD) with little or no side effect to the surrounding normal cells. Moreover, NIR-PIT can generate an immune response in distant metastases and inhibit further cancer attack by combing cancer cells targeting NIR-PIT and immune regulatory cells targeting NIR-PIT or other cancer treatment modalities. Several recent improvements in NIR-PIT have been explored such as catheter-driven NIR light delivery, real-time monitoring of cancer, and the development of new target molecule, leading to NIR-PIT being considered as a promising cancer therapy. In this review, we discuss the progress of NIR-PIT, their mechanism and design strategies for cancer treatment. Furthermore, the overall possible targeting molecules for NIR-PIT with their application for cancer treatment are briefly summarised.

## 1. Introduction

Cancer is one of the top global health problems. The primary cancer treatments are surgery, chemotherapy, and/or radiation, which are well known to cause considerable damage to normal cells. Over the last three decades, several newly developed modalities have been developed for cancer treatment including immunotherapy, targeted therapy, photoimmunotherapy (PIT), photothermal therapy, and photodynamic therapy (PDT) [1,2,3,4]. These treatment modalities aim to mitigate the cancer burden with fewer side effects. PDT is a medical treatment that mostly depends on the generation of cytotoxic singlet oxygen and other reactive oxygen species (ROS) that directly destroy tumor cells via the activation of a photosensitizer (Ps) by light irradiation at a corresponding wavelength [5,6,7]. Over the last 40 years, PDT has been extensively used to treat cancer cells, and several approaches have been developed to increase PDT activity and specificity. One of these approaches is the use of nanoparticle as a Ps delivery vehicle. Nanoparticle-based PDT takes advantage of the enhanced permeability and retention (EPR) effect, and thus has the potential for better Ps accumulation into tumor tissues and less side effects in the surrounding cells compared with conventional PDT [6]. Moreover, the incapability of destroying deep tumors using PDT can be overcome by applying fiber optic inserted interstitial photodynamic therapy (iPDT) [8]. PIT emerges as a novel promising approach, which is a combination of phototherapy and immunotherapy that leads to effective and specific eradication of primary tumors and distant metastasis, and that inhibits cancer recurrence [9]. Similar to PDT, PIT approaches kill the treated cells through the local activation of Ps with an appropriate light wavelength, but they have the advantages of a higher efficacy and less side effects due to the properties of the targeting moiety of PIT agents such as antibodies, peptides, and small ligands.

The fastest growing PIT approach in this field is the NIR-PIT approach. The cell death mechanism in NIR-PIT is distinctive, with features of cell swelling, the formation of bleb, and membrane rupturing, resulting in vigorous anti-tumor immune responses [10,11,12]. It has great potential to selectively and locally destroy cancer cells, inducing immunogenic cell death (ICD) and triggering an antitumor immune response [13]. This is mainly due to the release of damage-associated molecular patterns (DAMPs) and tumor-associated antigens (TAAs) after PIT, which activate antitumor immune responses [14,15]. However, this response is insufficient to treat the metastatic tumor as the immunosuppression of the tumor microenvironment could limit the antitumor immunity [3,16]. To induce the antitumor immune response, a combination of PIT with immune checkpoint inhibitors and immunoadjuvants were applied as a synergistic treatment against tumor cells [16]. NIR-PIT has advantages over conventional PDT as it does not depend on ROS generation, has a higher efficacy and little or no side effects on the surrounding cells [17].

For these reasons, NIR-PIT has gained encouraging success both in preclinical and clinical applications for different cancers [18]. Different targeting moieties were employed in NIR-PIT to enhance the tumor immunogenicity, targeting capability, stability, and flexibility of NIR-PIT agents [9,19,20,21].

## 2. Antibody and Antibody Mimetic-Based NIR-PIT

Tumor cell growth and progression are associated with either the up or down regulation of certain pathways, which in some cases requires an increased expression of related receptors. Therefore, targeting these receptors using specific ligands such as antibodies, their fragments, and mimetics can enhance the tumor cell selectivity and spare the surrounding healthy tissue [22]. In NIR-PIT, monoclonal antibodies and antibody derivatives such as fragment antigen-binding (Fab), F(ab′)_2_, single chain antibody fragment (scFv), diabody, minibody, and nanobody, as well as antibody mimetics, were used as delivery vehicles for the IRdye700 producing photo-immunoconjugates [23,24,25,26]. Several studies have demonstrated the success of photo-immunoconjugates using antibodies targeting receptors overexpressed in tumor cells [27].

Most clinical and preclinical studies involve cetuximab and panitumumab conjugated with IRdye700 (cetuximab-IRdye700 and panitumumab-IRdye700) for targeting the membrane receptor epidermal growth factor receptor (EGFR) overexpressing cancer cells [13,28]. After NIR light irradiation, cetuximab-NIR-PIT and panitumumab-NIR-PIT agents can selectively kill target cells and elicit host anti-tumor immune responses [28,29,30,31].

For homogeneous micro distribution of antibody-conjugates, a cocktail of panitumumab and basiliximab conjugated IRdye700 was used for NIR-PIT that significantly reduced tumor growth and prolonged the survival of tumor-bearing mice compared with the single antibody-IRdye700 conjugate [32]. When combining NIR-PIT reagents, panitumumab-IRdye700 and anti-CD25-F(ab′)_2_-IRdye700 demonstrated the effective inhibition of tumor growth and an enhanced antitumor immune response by depleting regulatory T (T_regs_) cells from the tumor microenvironment [33]. Furthermore, conjugating IRdye700 to pertuzumab and trastuzumab that targets human epidermal growth factor receptor 2 (Her2)-expressing cancer cells revealed antitumor effects after NIR light irradiation [25,34,35,36,37]. In addition, other Ps, for instance porphyrin and chlorin e6 conjugated trastuzumab, were also used to eliminate Her2 positive cancer cells with improvements in specific tissue access and penetration [38,39].

Besides targeting the EGFR and Her2, several antibodies targeting different TAAs have been used to generate NIR-PIT reagents. These include, for example, rituximab (anti-CD20) [40], anti-prostate-specific membrane antigen (PMSA) antibody [41], anti-epithelial cell adhesion molecule (EpCAM) antibody [42,43], anti-CD44 antibody [44], anti-CD47 antibody [45,46], and anti-tumor-associated calcium signal transducer 2 (TROP2) antibody [13,47,48]. Moreover, AC133 monoclonal antibody (mAb) [49] and anti-CD44 antibody [44,50,51,52] conjugated with IRdye700 agents were used for NIR-PIT against different cancers by targeting cancer stem cell marker AC133 and CD44, respectively.

In addition to the full-length mAb, their fragments have also been used to develop NIR-PIT reagents because of their smaller molecular size, more efficient tumor penetration, and faster tissue clearance. These fragments, such scFv and Fab, are lacking the antibody Fc region, which is responsible for initiating antibody-dependent cellular cytotoxicity (ADCC) and complement-dependent cytotoxicity (CDC) immune responses. For example, scFvs against EGFR [23,53], EpCAM, and chondroitin sulphate proteoglycan 4 (CSPG4) [54] were used to generate NIR-PIT agents. Furthermore, Sato et al. demonstrated that conjugating IRdye700 to F(ab′)_2_ targeting CD25 can selectively eliminate tumor-infiltrating T_regs_ [55]. A recent study reported that combining anti-CD44 antibody and anti-CD25 antibody conjugated with IRdye700 led to significant tumor growth inhibition and a prolonged survival rate [44]. Anti-CD25-IgG or its anti-CD25-F(ab′)_2_ derivative [44,55,56] and anti-cytotoxic T-lymphocyte-associated protein 4 (CTLA4) antibody [57] conjugated NIR-PIT agents can selectively eliminate the local T_regs_ only within the tumor microenvironment (TME), resulting in upregulation of the anti-tumor immune responses [47]. The effectiveness of NIR-PIT was demonstrated by using F(ab′)_2_ targeting programmed death-ligand 1 (PD-L1) and CD25 to generate anti-PD-L1-F(ab′)_2_-IRdye700 and anti-CD25-F(ab′)_2_-IRdye700, respectively [57,58]. Avelumab (human anti-PD-L1 mAb) [59,60] and F(ab′)_2_ fragments of the anti-PD-L1 antibody [60] conjugated with IRdye700 show significant therapeutic effects against different cancers.

A minibody named MS5 was generated by fusing its scFv to the human IgG1 Fc domain [61]. As MS5 can bind to solid and blood cancer cells, IRdye700 was conjugated to the MS5 and used for targeting various cancer cells. MS5-IRdye700 is able to kill target cells in both monolayer and tumor spheroid cultures by inducing the immunogenic cell death pathway [62]. Moreover, NIR-PIT targeting PSMA was generated by conjugating IRdye700 with anti-PMSA diabody and minibody. These PSMA-diabody-IRdye700 and PSMA-minibody-IRdye700 show significant inhibition of tumor growth and enhance survival rate [63]. Nanobodies (12–15 kDa) are antibody-fragments derived from heavy-chain antibodies, which are highly soluble and physically stable [64]. Nanobody targeting G protein-coupled receptor (GPCR) was site-specifically conjugated to IRdye700. This nanobody-IRdye700 selectively kills US28-expressing glioblastoma cells [65,66].

In addition, affibody molecules, which are small (6.5-kDa) affinity proteins that can bind protein targets with a high affinity and selectivity, were used to generate NIR-PIT [66,67]. Because of its small size, the affibody has rapid clearance, and good tumor penetration, and can even pass the blood–brain barrier [68]. Recently, the Her2 affibody (Z_Her2:2395_)-IRdye700 was shown to have selective cell death and trigger the release of all hallmarks of immunogenic cell death, resulting in the maturation of dendritic cell (DC) and the augmentation of anticancer immunity [69]. Another study demonstrated that IRdye700-Her2 affibody induced the necrotic cell death of Her2-overexpressing breast cancer cells [70]. In addition to the Her2 affibody, a dimeric platelet-derived growth factor receptor β (PDGFRβ) affibody (Z_PDGFRβ_) was conjugated with IRdye700 and showed the specific binding of PDGFRβ overexpressed pericytes of many types of tumors. After NIR light irradiation, Z_PDGFRβ_-IRdye700 specifically kills pericytes, resulting in damage to the tumor blood vessels, thereby inducing tumor destruction [71]. Furthermore, EGFR-specific affibody molecule (Z_EGFR:03115_) conjugated with IRdye700 shows brain tumor destruction by inducing ICD and turning an immunosuppressive tumor environment into an immune-vulnerable one [72,73]. The above-mentioned studies suggest that affibody-based NIR-PIT could be an attractive alternative to mAb-based NIR-PIT.

## 3. Peptide and Small Ligand-Based NIR-PIT

Besides antibodies, short peptide targeting TAAs were used to deliver IRdye700 specifically to the target cells. For example, IRdye700 was covalently conjugated to the arginine–glycine–aspartic acid (RGD) peptide linked with human serum albumin to target the integrins, which are highly expressed in several cancers. The nanoconjugate shows effective cancer-specific delivery and massive cancer cell killing [74]. The nanoconjugate is further developed by conjugating IRdye700 with the RGD peptide to 8-arm polyethylene glycol (PEG). The RGD–8PEG–IRdye700 demonstrates significant cancer cell reduction after light irradiation [75]. In addition, IRdye700 was conjugated to multiple cyclic RGD peptides (cRGD) to obtain better accumulation into the target sites with an increased number of cyclic RGD. The 15 cyclic RGD peptide polymer revealed significant inhibition of the tumor growth [76].

Low-molecular-weight (LMW) ligands are used also for targeting cancer antigens because of their efficient tumor penetration, reduced immunogenicity, and lack of Fc-mediated side effects [77,78]. A recent study demonstrated that the LMW ligand, IRdye700, targeting PSMA decreased cell viability via different cytotoxic mechanisms [79].

## 4. Therapeutic Mechanism of NIR-PIT

### 4.1. Physicochemical Therapeutic Activity

**Figure 1:**
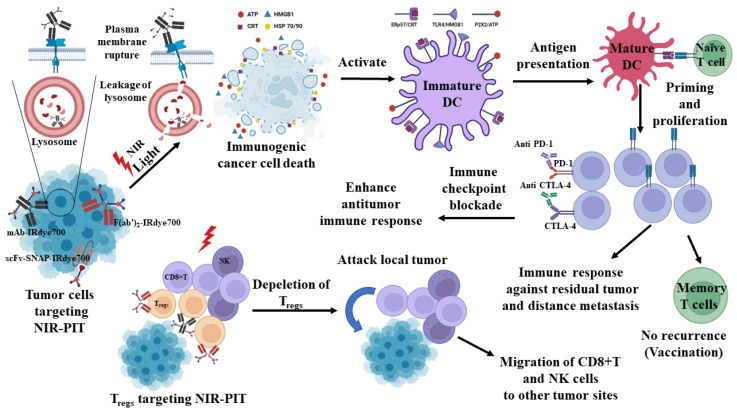
Mechanism of the NIR-PIT approach and its immunological consequences.

NIR-PIT induces cell death after NIR light treatment, which activates the photochemical ligand reaction that releases the hydrophobic side chain of IRdye700, which in turn makes the remaining molecule hydrophobic. This photochemical reaction changes the NIR-PIT bound to the cell membrane by forming water insoluble aggregates of NIR-PIT or a Z-stack multimer of silicon-phthalocyanine IRdye700 rings [80]. The reduction in the cell membrane integrity of the NIR-PIT-antigen complex can damage the target proteins, which causes immediate swelling of the cells and subsequently releases the intracellular materials [10,12,17,81]. Moreover, it is reported that NIR-PIT agents are aggregated on the plasma membrane and lead to necrotic cell death. Internalized NIR-PIT agents also have cytotoxic effects following NIR light irradiation. It can induce necrotic cell death via ROS generation, which causes significant leakage of the lysosomal contents into the cytosol (Figure 1). Nevertheless, cytotoxicity by NIR-PIT is weaker in lysosomes than in the plasma membranes [82].

### 4.2. ICD and Anti-Tumor Immune Augmentation

The chemical and physical damages associated with NIR-PIT lead to rapid disruption of the cell membrane, which is a characteristic feature of ICD. ICD is a local immune reaction initiated by releasing cancer antigens from the dying cancer cells [17,30]. NIR-PIT releases DAMPs hallmarks, such as calreticulin (CRT), high-mobility group box 1 (HMGB1), adenosine triphosphate (ATP), heat shock protein (Hsp) 70, and Hsp 90 [25,51]. These DAMPs markers are responsible for the maturation of DCs, which prime the naive CD8^+^ T cells. Primed CD8^+^ T cells are then proliferated due to the rapid release of multiple neoantigens and have the ability to attack the residual cancer cells (Figure 1). The induced immune cells could induce systemic anticancer immune response via their migration throughout the body and by attacking distant metastatic sites. In addition, host immunity might be enhanced by NIR-PIT targeting immune suppressor cells, resulting in the selective depletion of inhibitory immune cells [47] (Figure 1).

### 4.3. Super-Enhanced Permeability and Retention (SUPR)

NIR-PIT selectively kills the tumor cells without affecting healthy cells by targeting the overexpressed antigen on the target cells. The light treatment causes no damage to surrounding normal cells that lack the targeted antigen [25]. It is reported that the repeated administration of NIR-PIT and NIR light suppress residual cell growth and recurrence attacks [83]. After applying NIR light, the NIR-PIT bound perivascular cells undergo necrosis, generating a space between the vessels and the remaining tumor mass wall, which enhances the vascular permeability of the nanosized drug in the treated tumor bed. The nanotherapeutic agents can remain in the tumor bed for several days. This SUPR of NIR-PIT enables enhanced delivery of nanodrugs into the tumor bed. Several recent studies have shown that the therapeutic effect is more efficient when a combination of NIR-PIT and anticancer nano drugs are applied compared with corresponding therapies individually [33,44].

## 5. Designing Strategies for NIR-PIT

In general, NIR-PIT is generated by conjugating IRdye700 with tumor-specific ligands. Various strategies are reported for binding antibodies to IRdye700; however, the direct conjugation methods via lysine or cysteine residues of the antibody are the most commonly used methods [13]. These methods, also known as randomly conjugation methods, are associated with generating heterogeneous NIR-PIT agent populations resulting in an ununified therapeutic and safety profile of NIR-PIT [84]. Site-specific conjugation methods are applied to generate homogeneous NIR-PIT. One promising approach is based on exploiting the simple, controlled, and robust site-specific conjugation properties of the SNAP-tag conjugation method. Here, several scFv molecules were genetically fused with the SNAP-tag protein to allow for the conjugation of bengylguanine (BG) modified IRdye700 [54,85,86]. This approach generates highly homogenous NIR-PIT agents with unified pharmacokinetic properties. This study demonstrates the potent phototherapeutic activities of NIR-PIT against breast cancer, ovarian cancer, and skin cancer cells in vitro [23,53,54].

## 6. NIR Light Delivery Method for NIR-PIT

The delivery of NIR light to the target tissue is very crucial in order to achieve effective therapeutic activities. The penetration depth of NIR light into the tissue is approximately 2 cm from the surface [47]. NIR light can be applied with a conventional extracorporeal apparatus that has a frontal diffuser to the tumor site, for instance in the mouth and skin [87]. Nagaya et al. (2018) first described the efficacy of a fiber optic diffuser under endoscopic guidance that could deliver NIR light into the deeply located tumor, such as peritoneum and thorax tumors [88,89]. Recently, a novel catheter mounted with light emitting diodes (LEDs) was developed to overcome the limitations of conventional external irradiation devices and endoscope diffusers. This catheter has the ability of deep insertion and irradiation, non-kinking properties, and a temperature sensor that can assist in avoiding thermal burn. By using this catheter, tumor growth is significantly reduced in cholangiocarcinoma xenografts in mice treated with NIR-PIT [90]. More recently, an endovascular-therapy-based light illumination technology (ET-BLIT) was developed to provide deep light irradiation within the body. In ET-BLIT, a single lumen catheter system was used that contains a tip partial transparent catheter with a transparent distal end attached to the thermocouple head and an optical light diffuser selective for lateral light irradiation. By using ET-BLIT, the NIR light can reach the liver and kidneys without damaging the blood vessel or other side effects [91].

## 7. Monitoring the Therapeutic Effects of NIR-PIT

Real-time monitoring of the tumor accumulation, therapeutic response, and appropriate NIR light irradiation in NIR-PIT is important for precise treatment [47,92]. Several imaging methods are used to evaluate the effectiveness of NIR-PIT directly after treatment. Bioluminescence imaging (BLI) can be used preclinically to monitor the efficacy of NIR-PIT [93], but clinically, tumor cells cannot be transfected with the luciferase gene in advance [92]. One of the imaging methods during NIR-PIT is IRdye700 fluorescence imaging to confirm the accumulation of NIR-PIT agents in tumor tissues. The fluorescence signal of IRdye700 disappears after NIR light irradiation at 690 nm because the photochemical ligand-release reaction causes the precipitation of conjugated proteins [17,80,92]. As the glucose uptake of cancer cells increases dramatically, 18F-fluorodeoxyglucose positron emission tomography (18F-FDG PET) imaging provides early metabolic changes in the tumors, serving as an excellent method for evaluating the immediate treatment success in NIR-PIT-treated tumors. The 18F-FDG PET can be sustained for at least 24 h [94].

In addition to BLI and fluorescence imaging, optical coherence tomography (OCT) is used as a biomedical imaging tool that reveals dramatic hemodynamic changes in tumor vessels during NIR-PIT. This imaging system shows a significant difference in treated tumors compared with untreated tumors [95]. Moreover, the micro-distribution of the NIR-PIT agent from the tumor surface to the deep tumor during and after treatment and its therapeutic effects is monitored using a two-channel fluorescence fiber imaging system and two-photon microscopy with and without a microprism [96].

Recently, a customized camera system (LIGHTVISION) was designed to detect indocyanine green, and it can also detect the fluorescence arising from IRdye700 during NIR-PIT, which is implemented for intraoperative imaging of NIR-PIT. This camera system can monitor the NIR-PIT in real-time at wavelengths of 830 nm, which is far from the intense laser excitation light at 690 nm [97]. In addition, magnetic resonance imaging (MRI) is a widely used imaging modality, which can also be used to detect early therapeutic effects after NIR-PIT. The tumoricidal effects and hemodynamic changes induced by NIR-PIT can be monitored by ^13^C MRI, BOLD MRI and photoacoustic imaging [92,98].

## 8. Targeting Molecules for NIR-PIT and Their Application for Cancer Treatment

In the past few years, many NIR-PIT agents were developed for targeting different types of cancers. Initially, NIR-PIT was developed for targeting EGFR and Her2, and then expanded to target diverse cell surface proteins (such as EpCAM, CD44, CD47, CD25, PD-L1, and CTLA4) by using th mAb, antibody fragment, and nanobody. However, NIR-PIT can be applied to any cancer or regulatory cell in the tumor microenvironment if the available tumor antigens are overexpressed [47]. Here, we briefly summarized recently developed NIR-PIT agents (Table 1), as well as their clinical and preclinical applications.

### 8.1. EGFR

EGFR overexpression is observed in numerous solid tumors, including head-and-neck, prostate, ovarian, breast, renal, colon, lung, pancreas, skin, and esophageal cancers [33,47,133,134]. Therefore, EGFR is considered to be an excellent target for NIR-PIT and its therapeutic properties have been investigated by several studies [23,33,53,54,72,83,99,100,135,136]. In 2020, the first NIR-PIT drug (cetuximab-IRdye700) was conditionally registered and approved for clinical use in Japan [47].

### 8.2. Her2

Her2, a membrane tyrosine kinase, has been an established therapeutic target for several years and is overexpressed in different cancer cells including breast cancer [137], gastric cancer [138], and esophageal cancer [139]. Her2-targeted NIR-PIT has exhibited an effective cytotoxicity and significant reduction in tumor volume in Her2 expressing breast cancer [25], lung metastasis [36], non-small cell lung carcinoma [102], and ovarian cancer [101] both in vitro and in vivo. A more recent study showed that Her2 targeting NIR-PIT is more effective in cisplatin (CDDP) chemoresistance small cell lung cancer because Her2 expression is upregulated due to CDDP resistance [103]. In addition, a combination of Her2-targeted NIR-PIT combined with chemotherapy (5-fluorouracil) significantly reduced tumor growth in gastric cancer by inducing necrotic and apoptotic cell death [140].

### 8.3. PSMA and CEA

The overexpression of the membrane protein PSMA provided an efficient target for cancer treatment. Anti-PSMA-IRdye700 has been employed in NIR-PIT as a promising candidate for the treatment of PSMA-expressing prostate tumor [41], resulting in significant inhibition and prolonged survival of prostate tumor bearing mice. Several NIR-PIT studies were also performed in gastric and pancreatic cancer by targeting CEA, which showed a significant suppression of tumor growth and an improved survival rate [108,109,141,142].

### 8.4. CD44

CD44 is a well-characterized cancer stem cell marker [143] used as a potential therapeutic target for NIR-PIT. NIR-PIT with anti-CD44-IRdye700 has shown decreased tumor growth and an increased survival rate in a CD44 expressing oral cancer bearing mouse model [50]. Additionally, CD44-targeted NIR-PIT combined with immune checkpoint inhibitor (PD-1 or CTLA4) decreased tumor progression and significantly prolonged survival rate compared with single therapies in colon and lung tumors [51,52,60,106]. Tumor growth inhibition, prolonged survival, and increased anti-tumor immunity have been observed after combined treatment of CD44-targeted NIR-PIT and short-term IL-15 administration compared with the single-agent therapy in colon, lung, and oral tumor bearing subcutaneous mouse models [105].

### 8.5. PD-L1

PD-L1 is a transmembrane protein, and is overexpressed in numerous cancer cells including ovarian cancer, renal cell carcinoma, and melanoma. PD-L1 induces T cell tolerance by binding to PD-1, expressed on tumor-infiltrating lymphocytes [144]. Tumor growth was significantly inhibited after in vitro NIR-PIT treatment using anti-PD-L1-IRdye700 in a papillary adenocarcinoma bearing mouse model [60]. PD-L1 targeted NIR-PIT also eliminated PD-L1 expressing TAMs and cancer cells in ovarian cancer xenografts [59]. In addition, PD-L1 targeted NIR-PIT resulted in the depletion of the tumor and augmentation of the antitumor immunity in syngeneic mouse tumor. Although a limited cytotoxic effect was found in the in vitro study, an enhanced antitumor effect was observed in the colon, lung, and prostate tumor mouse models [58].

### 8.6. CTLA4

CTLA4-targeted NIR-PIT depletes intratumorally CTLA4 expressing T_regs_, resulting in an enhanced T cell mediated antitumor immunity. This local depletion of T_regs_ cells in the tumor bed combined with cancer targeting NIR-PIT is a promising antitumor therapy [57]. Combining NIR-PIT targeting CTLA4 expressing cells and cancer cells is highly effective at suppressing tumor growth and the depletion of CTLA4 expressing T_regs_. Thus, the antitumor immune response in distant untreated tumors is enhanced [128].

### 8.7. CD25

Successful cancer therapy is hindered by expanding tumor induced T_regs_ cells. Tumor-infiltrating CD25^+^ T_reg_ cell depletion was contemplated as a vital step for enhancing the anticancer immunity [145]. Local CD25 targeted NIR-PIT selectively eradicated intratumorally T_regs_, resulting in rapid activation of CD8 T and NK cells, leading to cell-mediated cancer killing [55]. Both antibody fragments and full-length antibody generated NIR-PIT (anti-CD25-F(ab′)_2_-IRdye700 and anti-CD25-IgG-IRdye700) targeting CD25 were able to significantly inhibit tumor growth in mice with the superior effect of anti-CD25-F(ab′)_2_-NIR-PIT [56]. Combining CD25-targeted NIR-PIT with CD44 or hEGFR-targeted NIR-PIT showed an improvement in tumor growth inhibition and prolonged survival in tumors compared with the single therapeutic agents [33,44].

### 8.8. Others

Several TAAs are targeted by NIR-PIT, including CD206, CD47, CD20, vascular endothelial growth factor receptor-2 (VEGF2), and fibroblast activation protein (FAP). TAM-targeted NIR-PIT using an anti-CD206 antibody inhibited the progression of a subcutaneous tumor [127]. NIR-PIT utilizing anti-VEGF2-IRdye700 selectively damaged the tumor vascular endothelium, exhibiting antitumor effects by angiogenesis inhibition in gastric cancer [126]. Repeated CD47-targeted NIR-PIT further suppressed tumor growth and improved survival compared with a single round of treatment [45,46]. FAP-targeted NIR-PIT suppressed tumor growth and reduced chemoresistance in an esophageal tumor model [107,146]. Another study demonstrated that FAP-α- targeted NIR-PIT was able to kill engineered cancer cells effectively and specifically [147]. Moreover, arming anti-CD20 mAb (rituximab) with IRdye700 led to significant inhibition of B-cell lymphoma growth [40].

### 8.9. Artificially Induced Antigens Targeting NIR-PIT

NIR-PIT relies on targeting highly expressed cell surface antigens, which are not expressed homogenously in all cancer types. Therefore, artificially induced antigen expression for targeting NIR-PIT could be an alternative approach. Yoshida et al. (2012) expressed exogenous Her2 extracellular domain (Her2-ECD) expression on Her2-negative breast cancer cells by transducing the Her2-ECD gene containing the adenoviral vector [148]. Her2-targeted NIR-PIT showed destruction of the Her2-negative breast cancer cells, inhibition of metastasis, and prolongation of survival in the generated cells in vivo [148,149,150]. A very recent study revealed that ephrin type-A receptor 2 (EphA2) encoding lentiviral vector was transduced to stably express EphA2 for EphA2 targeting NIR-PIT. The anti-EphA2-IRdye700 exhibited notable tumor growth inhibition in EphA2 transduced tumor cells. In addition, EphA2 targeted NIR-PIT elicited innate and adaptive immune responses within the treated tumor [151].

## 9. Limitation of NIR-PIT

Applying a single PIT agent with mAb against TAAs is unable to fully cure cancer. Moreover, the recurrence of cancer has also been observed after single PIT agent treatment. Multiple NIR-PIT targeting different TAAs in a broad range of cancer types is complicated, expensive, time-consuming, and unpractical. To generate versatile NIR-PIT agents, Shirasu et al. (2019) first developed novel NIR-PIT agents by combining biotinylated antibodies (BioAbs) with AvIR (IRdye700-conjugated NeutrAvidin) to facilitate unlimited target specificities. This approach can overcome the limitation of the repetitive preparation of IRdye700-mAb conjugates [152].

The recurrence of cancer attack in NIR-PIT-treated mice occurs because of the inhomogeneous intratumoral distribution of mAb-IRdye700 due to its relatively large molecular size. To overcome this limitation, systematic repeated NIR light exposure was administrated to the cancer cells in a fractionated manner of mAb-IRdye700, under the guidance of IRdye700 fluorescence signal [83]. Alternatively, a second mAb with a lower affinity was added to overcome the “binding site barrier” phenomenon, thereby facilitating the penetration of the second mAb into deep tumor cells [32].

The labelling of tumor-specific ligands with IRdye700 is generally associated with several drawbacks including, free IRdye700 impurities, heterogenous IRdye700-ligand conjugates from batch to batch, poor reproducibility, and aggregation [13,153,154]. Moreover, direct conjugation of ligands with IRdye700 may occur at the recognition site, which can hamper or even lose the targeting activity [13]. NIR-PIT relies on specific ligands for targeting highly expressed cell surface antigens, which are not always overexpressed in continuously developing cancers. Thereby, this technology is limited to tumors that express a high level of cell surface antigens [18]. However, a recent study demonstrated that NIR-PIT targeting low-expressed PD-L1 generated sufficient antitumor effects in tumor cells and an increased antitumor immunity [58].

The limitation of NIR-PIT also includes the difficulty of delivering NIR light to large tumor masses and to deeply located tumors such as lung tumor, intraperitoneal cancers, and cholangiocarcinoma [37,38,51,99,155]. Recently, an ET-BLIT was developed for light irradiation that can be accessible in the whole body [91]. Moreover, realistic, immunocompetent mouse models suitable for human diseases are required for studying the systemic antitumor immune responses [18,119,156]. As the growth of tumors is faster in mice than humans [157], no apparent side effects of repeated NIR-PIT were observed in a mouse model. In addition, the long term side effects of repeated NIR-PIT were not investigated [36]. A very recent study demonstrated that light doses at 100 J/cm^2^ could cause extensive local edema and weight loss [158].

NIR-PIT is mostly unable to induce durable antitumor responses due to the adaptive immune resistance of humans [51]. The single NIR-PIT agent was reported to be effective at killing cancer cells, but tumors can develop resistance to monotherapy [152]. To overcome this limitation, a combination of immune-checkpoint inhibitors [52,108], multiple NIR-PIT agents targeting different antigens [100], and T_regs_ targeting additional NIR-PIT agents [44] can be applied. NIR-PIT targeting T_reg_ cell antigens show tumor regression by depleting intratumoral T_reg_ cells. However, the activated T cells can also be depleted by ADCC and CDC due to the Fc region of the antibodies used in NIR-PIT [47]. Systemic depletion of T_regs_ may also induce adverse autoimmune events, which can be overcome by depleting the T_reg_ cells locally without eliminating T_regs_ in other organs [55,56].

## 10. Conclusions

NIR-PIT has become a promising treatment modality for any type of cancer by targeting cancer cells, immune-regulatory cells, or both, where NIR can reach. NIR-PIT has advantages over traditional therapy by eliminating the cancer cells in a highly specific manner and enhancing the host immune response with minimal off-target effects. When used in combination with immune-suppressor targeted NIR-PIT or adjuvant therapies, NIR-PIT can generate a robust antitumor immune response. The development of a new NIR light delivery system, uniform NIR light treatment methods, and real-time monitoring of the treatment effect has made NIR-PIT a potential treatment for a wide variety of cancers.

## Figures and Tables

**Table 1:** NIR-PIT agents and their clinical and preclinical applications.

Cancer Type	Target Molecule	Photo-Immunoconjugates	Therapeutic Outcome	References
Head and neck cancer, skin cancer, lung cancer, breast cancer, uterine cervical cancer, glioblastoma, bladder cancer	EGFR	* Cetuximab-IRdye700	TVR, ProS, AIA	[30,19]
		* Cetuximab-IRdye700 + anti-PD-L1	-	[19]
		scFv-425-SNAP-IRdye700	CCE	[53]
		Affibody (Z_EGFR:03115_)-IRdye700	CCE	[72]
		Panitumab-IRdye700	TVR, ProS	[99]
		Panitumab-IRdye700 + Trastuzumab-IRdye700	TVR	[100]
		Panitumab-IRdye700 + anti-CD25-F(ab′)_2_-IRdye700	TVR, ProS, AIA, IMD	[33]
Gastric cancer, breast cancer, lung cancer	Her2	Trastuzumab-IRdye700 Trastuzumab-emtansine-IRdye700	TVR, ProS Cytotoxic photo-bystander effect	[25,36,101,102,103,104]
		Affibody (Z_Her2:2395)_-IRdye700	TVR, AIA	[69]
Colon cancer, lung cancer, head and neck cancer, oral cancer	CD44	Anti-CD44-IRdye700 + Interleukin-15	TVR, ProS, AIA	[105]
		Anti-CD44-IRdye700 + anti-CTLA4-mAb	TVR, ProS	[106]
		Anti-CD44-IRdye700 + anti-PD-1	TVR, ProS, AIA	[51,52]
Brain cancer	CD133	AC133 mAb-IRdye700	TVR, ProS	[49]
Esophageal cancer	CAFs	Anti-FAP-IRdye700 + 5-fluorouracil (FU)	CCE	[107]
Prostate cancer	PSMA	Anti-PSMA mAb-IRdye700	TVR, ProS	[41]
Colon cancer, biliary tract cancer, pancreatic cancer	CEA	Anti-human CEA mAb-IRdye700	TVR	[108,109]
Lymphoma	CD20	Rituximab-IRdye700	TVR, ProS	[40]
Colon cancer, gastric cancer, pancreatic cancer	TROP2	Humanized anti-TROP2-IRdye700	TVR	[48]
Melanoma	CD146	Anti-CD146 mAb (YY146)-IRdye700	TVR	[110]
Colorectal cancer	GPA33	Anti-GPA33-scFv-IRdye700	TVR	[111]
Hepatocellular carcinoma	GPC3	Anti-GPC3-IRdye700 + nab-paclitaxel	TVR	[112]
Lung cancer	DLL3	Rovalpituzumab-IRdye700	TVR, ProS	[113]
Gastrointestinal cancer	Cadherin-17	Anti-cadherin-17 mAb-IRdye700	TVR	[114]
Gastrointestinal cancer	c-KIT	Anti-c-KIT-IRdye700	TVR	[115]
Mesothelioma	PDPN	Anti-PDPN mAb-IRdye700	TVR, ProS, AIA	[116]
Mesothelioma, pancreatic cancer, ovarian cancer	Mesothelin	Anti-mesothelin-IRdye700	TVR, ProS	[117]
Melanoma	CD29	Anti-CD29-IRdye700 + anti-CD44- IRdye700 and anti-CTLA4 mAb	TVR, ProS	[118]
Lung cancer, mesothelioma	GPR87	Anti-GPR87-IRdye700	TVR	[119]
Prostate, bladder, brain, ovarian cancer	GPC-1	Miltuximab-IRdye700	TVR	[120]
Breast cancer	ICAM-1	Anti-ICAM-1-IRdye700	TVR, ProS	[121]
Prostate and breast tumor	JAM-A	Anti-JAM-A mAb-IRdye700	Decreases number of mitotic cells in cancer tissue	[122]
Lung cancer	CD276	Anti-CD276-F(ab)-IRdye700 + anti-PD-1/PD-L1 blockade	TVR, ProS, AIA	[123]
Colorectal cancer	PDGFRβ	PDGFRβ affibody (Z_PDGFRβ_)-IRdye700	Killed pericytes, damaged tumor blood vessels, antitumor effect	[71]
Lung cancer	MRP1	Anti-MRP1 mAb-IRdye700	TVR	[124]
Lymphoma	CLA	Anti-CLA-IRdye700	AIA	[125]
Chemoresistant tumors	Pgp	Anti-Pgp mAb-IRdye700 or Anti-Pgp-F(ab)-IRdye700	TVR, ProS	[126]
Gastric cancer	VEGFR-2	Anti-VEGFR-2 mAb-IRdye700	Damage in tumor neovasculature	[35]
Lung cancer, ovarian cancer	PD-L1	Avelumab-IRdye700	TVR, ProS AIA, AAE	[59,60]
		Anti-PD-L1-F(ab)_2_-IRdye 700	TVR, ProS	[58]
Colon cancer, lung cancer, prostate cancer, head and neck cancer	CD25	Anti-CD25 mAb-IRdye700 or anti-CD25-F(ab′)_2_-IRdye700	TD, AIA	[55]
		Anti-CD25 mAb-IRdye700 + anti-CD44 mAb-IRdye700	TVR, ProS, AIA	[44]
Breast cancer, lung cancer	CD206	Anti-CD206 mAb-IRdye700	Suppressed sorafenib-resistant tumors	[127]
Colon cancer, lung cancer, head and neck cancer	CTLA4	Anti-CTLA4-IRdye700	TVR, ProS, TD, AIA	[57]
		Anti-CTLA4-IRdye700 + anti-hEGFR-IRdye700	TVR, ProS, TD, AIA	[128]
		Anti-CTLA4-IgG-IRdye700 or anti-CTLA4-F(ab′)_2_-IRdye700	TVR, ProS, TD, AIA	[129]
Colon cancer, lung cancer	VISTA	Anti-VISTA mAb-IRdye700	TVR, ProS	[130]
Breast cancer	*Gr-1*	Anti-GR-1-IRdye700	Eliminate tumor-induced splenic MDSCs	[131]
Oral cancers, colon cancer	Ly6G	Anti-Ly6G mAb-IRdye700 + Panitumab-IRdye700	Eliminate MDSCs, TVR, ProS, AAE	[132]

*, Clinical trials in human; EGFR, epidermal growth factor receptor; Her2, human epidermal growth factor receptor-2; CAFs, cancer-associated fibroblasts; PSMA, prostate-specific membrane antigen; CEA, carcinoembryonic antigen; TROP2, tumor-associated calcium signal transducer 2; GPA33, glycoprotein A33 antigen; GPC3, Glypican-3; DLL3, delta-like protein 3; PDPN, podoplanin; GPR87, G-protein receptor; GPC-1, glypican-1; ICAM-1, intercellular adhesion molecule-1; JAM-A, junctional adhesion molecule-A; PDGFRβ, platelet-derived growth factor receptor β; MRP1, multidrug resistance protein 1; CLA, cutaneous lymphocyte antigen; Pgp, P-glycoprotein; VEGFR-2, vascular endothelial growth factor receptor 2; PD-L1, programmed death-ligand 1; CTLA4, cytotoxic T-lymphocyte-associated protein 4; VISTA, V-domain immunoglobulin suppressor of T cell activation; *GR-1*, granulocyte receptor-1 antigen; Ly6G, lymphocyte antigen 6 complex locus G6D; TVR, tumor volume reduction; ProS, prolonged survival; CCE, cancer cell elimination; AIA, antitumor immune augmentation; TD, T_regs_ depletion; AAE, abscopal antitumor effect; IMD, immunologic memory development; MDSCs, myeloid-derived suppressor cells.

## Data Availability

Not applicable.
